# The impact of dementia on drug costs in older people: results from the SNAC study

**DOI:** 10.1186/s12883-016-0547-x

**Published:** 2016-02-29

**Authors:** Anders Sköldunger, Johan Fastbom, Anders Wimo, Laura Fratiglioni, Kristina Johnell

**Affiliations:** Aging Research Center, Center for Alzheimer Research, Department of Neurobiology, Care Sciences and Society, Karolinska Institutet and Stockholm University, Stockholm, Sweden; Stockholm Gerontology Research Center, Stockholm, Sweden; Division of Neurogeriatrics, Center for Alzheimer Research, Department of Neurobiology, Care Sciences and Society, Karolinska Institutet, Huddinge, Sweden; Bollebergsvägen, 5, 820 70 Bergsjö, Sweden

**Keywords:** Costs, Dementia, Drugs, Generalized linear model, Health economy, Pharmacoeconomics, Population-based study

## Abstract

**Background:**

We aimed to investigate the impact of dementia on drug costs in older people, after adjustment for socio-demographic factors, residential setting and co-morbidities.

**Methods:**

We included 4 129 individuals aged ≥ 60 years from The Swedish National Study on Aging and Care (SNAC) in Kungsholmen and Nordanstig 2001–2004. A generalized linear model (GLM) was used to investigate how much dementia was associated with drug costs.

**Results:**

Overall drug costs for persons with and without dementia were 6147 SEK (816 USD) and 3810 SEK (506 USD), respectively. The highest drug cost was observed for nervous system drugs among persons with dementia. The adjusted GLM showed that dementia was not associated with higher overall drug costs (β = 1.119; ns). Comorbidities and residential setting were the most important factors for overall drug costs.

**Conclusion:**

We found that the observed higher overall drug costs for persons with dementia were due to comorbidities and residential setting.

## Background

Worldwide, more people reach old age as life expectancy continues to increase [1]. The aging of the population entails challenges for the health care system and for resource allocation. One of the most important challenges is the expected increase in number of people with dementia. This detrimental condition causes great suffering for the affected individuals and their families as well as immense costs for the society [2–4].

Another important challenge is the extensive use of drugs among older people [5], which accounts for the majority of societal drug expenditures [6]. With aging come changes in both the pharmacodynamics and pharmacokinetics of drugs, which leads to a higher sensitivity to drugs and susceptibility to adverse drug reactions [7]. Indeed, adverse drug events in older people entail significant costs in terms of care and hospitalizations [8]. A part of this problem is also comorbid conditions which are often present in the older people [9]. Particularly vulnerable are persons with dementia, in whom the neurodegenerative processes lead to a higher sensitivity to central nervous system (CNS)-acting drugs. Nonetheless, use of psychotropic drugs is very common among persons with dementia [10], although these drugs have been related to serious adverse outcomes in this frail group [11–13]. Drugs have been reported to account for about 2 % of the total costs for dementia [2]. However, new drug therapies emerge and in the future we may be able to treat dementia patients with disease modifying drugs, which will most certainly be very costly [14].

Research on drug use as well as drug costs in dementia is important from a resource allocation perspective. However, research about costs of drugs among frail persons with dementia and older people in general is scarce. Many studies were conducted several years ago when today’s widely prescribed drugs, such as anti-dementia drugs, were not yet implemented in clinical practice [15]. In addition, most of these previous studies only analyzed overall drug costs and not individual drug classes.

Residential setting is an important factor for both drug use and dementia status [5]. People living in institutional settings use on average almost twice as many drugs as people living at home [5]. Moreover, since people with dementia who live in institutions are more cognitively impaired than their community-dwelling counterparts [10] their susceptibility to side effects are even more profound and residential setting should therefore be accounted for in analyses of drug use in dementia.

Thus, we aimed to investigate whether dementia was associated with drug costs in older people.

## Methods

### Study population

The Swedish National Study on Aging and Care (SNAC) is an ongoing, population based, longitudinal study of aging and health conducted at four different sites in Sweden. We analyzed data from the baseline examination conducted in 2001–2004 from Nordanstig in the middle part of Sweden and from Kungsholmen/Essingeöarna in the central part of Stockholm. Inclusion criteria were having an address in either of the actual areas at time of birthday for the ages specified below.

The SNAC study has been described in detail elsewhere [16]. In short, people aged 60, 66, 72, 78, 81, 84, 87 and ≥90 years are interviewed by a nurse about a wide range of domains including socioeconomic status, living habits and family history. Participants are also examined by a physician, memory tested by a psychologist and laboratory tests are collected. Data about diseases and drug use are collected during the interview with the physician. When the participant is not able to provide information, a relative is asked instead. If the person lives in an institution, the information is most often collected from medical records and staff.

### The care system for older people in Sweden

In Sweden, care for the elderly – and the associated costs – are divided between municipalities and the county council. Social care (e.g. home services, long term institutional care and day care) is covered by the municipalities while primary health care and specialist care are organized by county councils. Individual drug expenditure is to a great extent subsidized in Sweden. In 2003, the maximum level of out of pocket expense for drugs was 1,800 SEK per 12 month period. Overall, the majority of costs for social and medical care in Sweden are publically funded by taxes.

### Definitions

#### Socio-demographic variables

Age was categorized into 60–69, 70–79, 80–89 and ≥90 years in the descriptive analysis and used as a continuous variable in the Generalized Linear Model (GLM). Residential setting was dichotomized into community-dwelling (i.e. living in one’s own home) vs. institution (sheltered accommodation, old people’s home, group dwelling or nursing home). Educational level was classified into elementary vs. additional schooling.

### Dementia diagnosis

Dementia was diagnosed by physicians according to Diagnostic and Statistical Manual of Mental Disorders DSM III-R [17].

### Drugs

Drug data were self-reported (or reported by a proxy) [10]. Participants were asked to bring a list of currently used drugs to the interview. Prescriptions and medical containers were also inspected when available. Drugs were classified according to their Anatomical Therapeutic Chemical (ATC) code, as recommended by the World Health Organization (WHO) [18].

Drug costs were calculated based on a register with drug prices from the National Corporation of Swedish Pharmacies (Apoteket AB) from 2003. Each drug of each subject was sought out in the drug register. Thereafter, a matching preparation and strength was looked up. Among the records in the register that fulfilled these criteria a suitable package was selected. For tablets or capsules, packages with 100 or close to 100 tablets/capsules were selected. For other preparations, such as mixtures, the largest package was selected. The price of the package was divided by the number of units; for example number of tablets/capsules, or number of ml for liquid preparations. The obtained price per unit was then multiplied with the number of units taken daily by the subject. For drugs taken as needed we instead calculated the price per Defined Daily Dose (DDD), which is the average daily dose of a drug when used for its main indication in an average 70 kg adult, as established by WHO, and we assumed that as needed drugs were taken in an average dose of half a DDD per day. For anti-infective drugs, we assumed a limited treatment period of 20 days/year [15]. The costs are expressed in the Swedish currency SEK. In December 2003, 100 SEK corresponded to 13.27 USD. The results are mainly presented as costs per individual, which means the total cost for a drug or drug group in a group of elderly people, divided by the total number of subjects in that group. If the cost is instead presented per user, then that is noted in the text.

### Comorbidity

A modified Charlson Comorbidity Index was used to adjust for confounding by co-morbidities [19, 20]. The index contains seven diagnoses with the weight of one (myocardial infarction, congestive heart failure, cerebrovascular disease, dementia, chronic pulmonary disease, connective tissue disorder and diabetes without complication) and two diagnoses with the weight of two (moderate or severe renal failure and any tumour), which gives a total of maximum eleven. The comorbidity index was entered as a categorical variable in the analysis (none, one, two and three or more modified Charlson index weights). All diagnoses except renal failure and dementia diagnosis were based on medical history and examination of the physician. Renal failure was calculated from Cockgroft-Gaults formula [21] and defined as creatinine clearance < 25 ml/min. The dementia diagnosis procedure is described above.

### Physical functioning

Physical functioning was assessed by the Katz index of Activities of Daily Living (ADL) [22], which is a scale that measures dependency in six basic activities: bathing, dressing, going to the toilet, transferring, feeding and continence. Functional independence was defined as no need of assistance, partially dependent was defined as being dependent in one or two activities and being dependent was defined as dependent in three or more activities.

### Statistical analysis

The cost data had a non-normal and skewed distribution. Accordingly, regression analysis of costs was performed by using a generalized linear model (GLM) with the assumption of a gamma shaped distribution of the dependent variable [23]. GLMs are generally well suited for statistical analysis of cost data, which often show a high degree of non-normality.

We used a two-step procedure. First, logistic regression with costs as binary outcome was performed in order to understand which of the factors that was associated with the highest costs. Second, a GLM model was run to explore the magnitude of the cost-driving factors. In the GLM, the cost driving factors were dichotomized and first entered separately. All models were adjusted for age, gender and education. In the joint analysis, all factors were entered simultaneously. Dementia diagnosis is included in the Charlson Comorbidity Index, but in the joint analysis, dementia was analyzed as a separate variable and, thus, was removed from the index. Results of the GLM are expressed as relative change in cost due to increase in the explanatory variable (e.g. dementia). Inclusion of site (i.e. Kungsholmen and Nordanstig) did not affect the results; therefore, this variable was not included in the analyses. IBM SPSS Statistics 22.0 for Windows (IBM corp., New York, NY, USA) was used for the analyses.

### Ethics

The study was approved by the ethical review board in Stockholm (dnr 01–114) and in Uppsala (dnr 01–123). All participants were informed about the study and thereafter gave a written informed consent, and if not possible a proxy (i.e. a spouse or next of kin) gave the consent.

## Results

Of the 4,129 participants, 21 did not have information on drug use and were therefore excluded from the analyses (*n* = 4,108). Mean age for people without dementia was 73.2 (SD 10.6) years and for people with dementia 88.1 (SD 7.2) years (Table 1) (*n* = 4,108). Community-dwelling participants had a mean age of 73.9 (SD 10.6) years and participants in institutions 88.6 (SD 7.5) years.

The mean number of drugs for persons with dementia was 5.4 and for people without dementia 3.5 drugs (*p* < 0.001). The mean number of drugs used among community-dwelling persons was 3.4 and in institutions 6.3 (*p* < 0.001).

**Table 1:** Study population demographics according to dementia status (n=4108)^a^

	Non-dementia	Dementia	p-value
	*n*= 3,783	n=319	
Gender, n (%)			
Men	1,462 (38.6)	63 (19.7)	<0.001
Women	2,321 (61.4)	256 (80.3)	< 0.001
Age groups, n (%)			
60-69	1,555 (41.1)	4 (1.3)	< 0.001
70-79	1,085 (28.7)	40 (12.5)	< 0.001
80-89	763 (20.2)	106 (33.2)	< 0.001
≥90	380 (10.0)	169 (53.0)	< 0.001
Residential setting, n (%)			
Community-dwelling	3,699 (97.8)	124 (38.9)	< 0.001
Institution	84 (2.2)	195 (61.1)	< 0.001
Education^b^, n (%)			
Elementary	941 (25.1)	156 (50.8)	< 0.001
Additional	2,823 (74.9)	153 (49.2)	< 0.001
Mean number of drugs	3.48	5.42	< 0.001

^a^ Missing data on dementia status on 5 persons, 1 person mentally retarded and excluded

^b^ Missing data on 29 persons

The overall drug costs are summarized in Table 2. The mean drug cost in the study population as a whole was close to 4,000 SEK per person and year (Table 2). The drug cost was about 60 % higher in elderly persons with dementia than in those without the disease and more than 80 % higher in institutions compared to own home. However, in institutions, elderly persons with dementia had lower drug costs than persons without dementia.

**Table 2:** Annual drug cost per person (SEK) stratified by dementia status and residential setting^a^

	Community-dwelling Drug cost (SEK)	Institution Drug cost (SEK)	All Drug cost (SEK)
Dementia	5,010	6,869	6,147
No dementia	3,733	7,190	3,810
All	3,772	6,963	3,991

^a^Missing data on 6 persons

Cardiovascular drugs (ATC group C), nervous system drugs (ATC group N) and drugs for the alimentary tract and metabolism (ATC group A) constituted the major part of the drug costs −55 % in elderly persons without and 73 % in elderly persons with dementia (Table 3). The cost of nervous system drugs was more than five times higher in persons with dementia than without (3,202 SEK vs 585 SEK). This was explained by the higher use of virtually all types of nervous system drugs, including analgesics, antiepileptics, psychotropics and anti-dementia drugs, among the persons with dementia. For example, antipsychotics were used by 2 % of persons without and 22 % of persons with dementia. Marked differences were also seen for opioids (non-dementia (ND): 7 %; dementia (D): 22 %), anxiolytics (ND: 6 %; D: 28 %), antidepressants (ND: 8 %; D: 30 %) and anti-dementia drugs (ND: 0.3 %; D: 12 %). In addition, some of the nervous system drugs that were mainly used by persons with dementia were expensive, particularly the anti-dementia drugs with a mean annual cost per user of 10,884 SEK.

**Table 3:** Use of drugs in the main ATC groups and the estimated annual drug cost per person in persons with and without dementia and annual drug cost per person stratified by residential setting

		Drug use (%)		Cost/year (SEK)		Drug use (%)		Cost/year (SEK)		Drug use (%)	Cost/year (SEK)
Main ATC Group		Non-dementia	Dementia	Non-dementia	Dementia	Community-dwelling	Institution	Community-dwelling	Institution	All	
		*n*=3,783^a^	*n*=319^b^			*n*=3,826	*n*=282			n=4,108	
A	Alimentary tract and metabolism	35.1	52.7	496	789	34.7	60.3	487	952	36.5	519
B	Blood and blood forming agents	29.5	47.3	330	215	29.6	48.2	315	394	30.9	320
C	Cardiovascular system	44.8	53.0	1,009	514	44.4	57.8	997	596	45.4	969
D	Dermatologicals	3.0	11.0	24	66	3.0	12.1	23	78	3.7	27
G	Genito urinary system and sex hormones	22.0	13.8	348	256	21.8	14.9	344	296	21.3	341
H	Systemic hormonal preparations, excl. sex hormones	12.5	14.1	57	40	12.3	15.6	56	54	12.6	56
J	General anti-infectives for systemic use	1.7	4.4	5	12	1.7	5.7	5	13	1.9	6
L	Antineoplastic and immunomodulating agents	2.3	1.3	245	94	3.0	1.1	245	68	2.2	233
M	Musculo-skeletal system	17.1	8.5	261	137	16.8	11.3	260	132	16.4	251
N	Nervous system	34.4	67.0	585	3,202	33.8	79.8	607	3,295	37.0	792
P	Antiparasitic products	0.6	1.6	127	458	0.7	1.4	121	584	0.7	152
R	Respiratory system	12.3	13.2	274	256	12.0	16.7	266	344	12.3	272
S	Sensory organs	6.7	8.4	47	108	6.6	11.7	44	155	6.9	52
V	Various	0.2	0	3	0	0.2	0	3	-	0.1	2
	Total*	81.7^c^	79.8^c^	3,810	6,147	80.9^c^	88.4^c^	3,773	6,961	81.4^c^	3,992

^a^ Missing drug data on 4 persons

^b^ Missing drug data on 2 persons

^c^ Use of at least one drug

Cardiovascular drugs showed a higher use but to a lower mean cost in persons with dementia (Table 3). This was because they predominantly used high-ceiling diuretics (ND: 13 %; D: 37 %), nitrates (ND: 9 %; D: 17 %) and cardiac glycosides (ND: 4 %; D: 16 %), while elderly without dementia had a higher use of beta blocking agents (ND: 21 %; D: 12 %), calcium channel blockers (ND: 7 %; D: 4 %), angiotensin II antagonists (ND: 4 %; D: 1 %) and lipid modifying agents (ND: 12 %; D: 1 %) – in particular the latter two being expensive (yearly cost of SEK 2,416 and 3,092 per user, respectively). Drugs for the alimentary tract and metabolism were more common and, proportionally, carried a higher cost in persons with dementia. This was mainly explained by a higher use of laxatives (ND: 7 %; D: 38 %) and drugs for peptic ulcer and gastro-oesophageal reflux disease (ND: 9 %; D: 14 %).

The analyses focusing on residential setting (Table 3) showed great differences in drug use and associated costs between the community-dwelling and institutional setting. Most drugs were more commonly used in institutions. This was most evident for dermatologicals, anti-infectives and nervous system drugs. In particular, the costs for nervous system drugs were more than five times higher in institutions. The opposite relationship was found for cardiovascular drugs where costs were more than 50 % higher in the community-dwelling setting.

Table 4 shows drug use and costs in persons with dementia stratified by residential setting. Virtually all drug groups were more common in institutions, particularly dermatologicals, anti-infectives, respiratory system and nervous system drugs. A similar pattern was observed for the drug costs.

**Table 4:** Use of drugs in the main ATC groups and the estimated annual drug cost per person in persons with dementia stratified by residential setting^a^

Main ATC group		Community-dwelling		Institution	
		(n=124^a^)		(n=195)	
		Use (%)	Cost SEK	Use (%)	Cost SEK
A	Alimentary tract and metabolism	41.3	569	59.5	929
B	Blood and blood forming agents	41.3	237	50.8	201
C	Cardiovascular system	42.8	578	59.0	473
D	Dermatologicals	4.7	24	14.9	94
G	Genito urinary system and sex hormones	12.7	297	14.4	230
H	Systemic hormonal preparations, excl. sex hormones	10.3	9	16.4	60
J	General anti-infectives for systemic use	0.8	1	6.7	18
L	Antineoplastic and immunomodulating agents	1.6	235	1.0	5
M	Musculo-skeletal system	6.3	136	9.7	137
N	Nervous system	44.4	2,291	81.0	3,782
P	Antiparasitic products	1.6	486	1.5	440
R	Respiratory system	5.5	89	17.1	362
S	Sensory organs	7.9	59	8.7	139
V	Various	-	-	-	
	Total^b^	66.9	5,011	88.7	6,869

^a^ Missing data on drugs for 2 persons

^b^ Use of at least one drug

The results of the GLM analysis (Table 5) shows that comorbidities were the strongest drug cost driver (exp β = 2.265; <0.001) followed by residential setting (exp β = 1.878; <0.001) and dementia (exp β = 1.628; <0.002), after adjustment for age, gender and education. The joint analysis showed the same pattern with comorbidities as the strongest cost driving factor. However, dementia was not associated with higher overall drug costs (exp β = 1.119; ns) in this fully adjusted model. Male gender was associated with higher costs for drugs in all models, although not significant in the model adjusted for comorbidities and in the joint analysis.

**Table 5:** Relative impact of influential factors on annual cost of drug use^a^

	Model 1		Model 2		Model 3		Model 4		Model 5	
	Functional dependency Exp(β)	p-value	Residential setting Exp(β)	p-value	Comorbidities Exp(β))	p-value	Dementia Exp (β)	p-value	Joint analysis Exp (β)	p-value
Intercept (SEK)	3,234	<0.001	3,248	<0.001	5,258	<0.001	3,241	<0.001	2,262	<0.001
Age	1.003	ns	1.003	ns	0.998	ns	1.003	ns	0.996	ns
Gender^b^	1.203	0.025	1.209	0.021	1.050	ns	1.208	0.024	1.057	ns
Education^c^	1.109	ns	1,103	ns	1.152	ns	1.119	ns	1.158	ns
Functional dependency^d^	1.570	0.001							1.121	ns
Residential setting^e^			1.878	<0.001					1.629	0.017
Comorbidities^e^					2.265	<0.001			2.194	<0.001
Dementia^e^							1.628	0.002	1.119	ns

Exp (β) is interpreted as the relative change in cost due to a one unit increase in the explanatory variable

^a^ Missing data on 41 persons

^b^Female gender reference

^c^ Elementary school reference

^d^ No functional dependence reference

^e^ No dementia reference

^f^ Community-dwelling reference

## Discussion

In our population-based study of older persons, people with dementia used more drugs and had higher overall drug costs than persons without dementia. However, after adjustment for comorbidities and residential setting, dementia was not associated with higher overall drug costs. This reflects the complex interplay between different cost driving factors in the frail elderly population.

Fastbom and Giron (1998) have previously reported that in the Swedish Kungsholmen-project Phase IV from 1994 to 1996 including people aged >80 years [15], people with dementia had only slightly higher drug costs than people without dementia (2,806 SEK vs. 2,539 SEK). Even though we now analyzed a younger age group, the costs of drugs have increased in the past 7 years, particularly in the dementia group (6,147 SEK for people with and 3,810 SEK for people without dementia) and this increase is not only explained by inflation. The higher costs in the dementia group are partly explained by the introduction of the cholinesterase inhibitors and memantine after the study by Fastbom and Giron, but also a markedly higher use of antidepressants, antipsychotics and opioids among people with dementia. Previous research has also found that opioids are more common in patients with dementia and in the nursing home setting, which may have implications for patient safety [24]. In addition, the mean number of drugs in older people has increased over time [25, 26]. We have recently reported that the mean cost for drugs in dementia patients was estimated to 5,065 SEK per person in a 10-year simulation study and that number represented about 2 % of the total cost per year for the care of dementia patients [2]. Compared to the simulated drug costs of 5,065 SEK, we report higher drug costs (6,147 SEK) in this population-based study. However, the figure of 5,065 SEK was based on a combination of a rather old cost study and purchase data of anti-dementia drugs. Thus, our present figure is more valid and rather confirms the magnitude of the cost presented in our previous study.

Our findings are in line with a US study that reported a greater overall annualized total drug cost for patients treated with Alzheimer’s drugs than that of patients not receiving these drugs [27]. The authors conclude that the higher drug costs among those using Alzheimer’s drugs was due to concomitant drug therapy associated with the condition, a finding that is similar to ours. To our knowledge, there are no previous studies that have analyzed drug costs in relation to dementia status in a regression model adjusted for background factors. With this approach, we obtained the interesting finding that the observed higher crude drug cost for persons with dementia was confounded by comorbidities and residential setting. This may be explained by the substantial use of nervous system drugs, which we observed both in elderly persons with dementia and in the institutional setting. Use of psychotropics is extensive among persons with dementia who live in nursing homes [28–31], although there are some encouraging evidence that prescribing of these drugs to frail older persons is declining [32]. Thus, our findings are in line with the conclusion of the US study [27] and indicate that the higher drug costs among elderly persons with dementia are not only due the specific therapy with anti-dementia drugs but also to concomitant drug therapy associated with the condition, i.e. nervous system drugs including psychotropics.

The patents for all cholinesterase inhibitors have expired since the time of data collection in 2001–2004 in Sweden. Therefore, today the price of these drugs is much lower than at time of baseline data collection for this study. However, the use of these drugs has also increased [33]. In our study, 12 % of persons with dementia used anti-dementia drugs, which is much lower than reported from more recent data [33]. Even though the incidence of dementia has recently been reported to decrease [34], we can expect an increase in numbers of people with dementia due to the aging of the population and increasing survival of patients with dementia. Hence, the costs for anti-dementia drugs are expected to increase in the future [35]. This is particularly the case if Disease Modifying Treatment (DMT) [36] will reach the market, since the cost of this treatment is expected to be more expensive [14].

Drugs belonging to the group alimentary tract and metabolism were more frequently used by people in institutions and in those with dementia. The associated costs were also higher. This was mainly explained by the high use of laxatives among these frail groups. Constipation and subsequent use of laxatives are common among frail elderly persons [5, 26] and are often side effects of psychotropic drugs and opioids [29, 37]. On the contrary, costs for cardiovascular drugs were lower in the dementia and institutionalized groups, even though the use was more frequent than among persons without dementia and those living in the community. We have previously reported that institutionalized elderly persons are less likely to use recommended cardiovascular drugs (i.e. angiotensin-converting enzyme (ACE) inhibitors, angiotensin II antagonists, calcium channel blockers and beta-blocking agents) and more likely to use high-ceiling diuretics [5] and this is supported by our present findings. This practice explains the lower costs of cardiovascular drugs among persons in institutions and with dementia. Persons with dementia were older and their cardiovascular drug treatment may have been initiated several years ago and then continued although new and more expensive cardiovascular drugs have reached the market [38]. This highlights the necessity of medication reviews in elderly people with and without dementia and in people living in institutions.

### Limitations

We analyzed cross-sectional data which does not allow us to establish temporal sequence and draw causal conclusions.

Overall response rate in Kungsholmen was about 73 % and in Nordanstig 75 %. There was no difference in the sex distribution between non-responders and responders in Kungsholmen, but in Nordanstig slightly more men participated (75 %) than women (73 %).

Self-reported data on drug use run the risk of recall bias. However, compared with prescription register data, we avoid the limitations of lack of information about secondary non-adherence and we could also include use of over-the-counter drugs. Our data were based on drugs that were actually taken, not only purchased. This might be regarded as a disadvantage in pharmacoeconomic evaluations because their focus is often on the actual payment for drugs. Therefore, our costs may be underestimated, particularly among older persons outside of the residential setting and without dementia because intake of drugs is not monitored to the same extent in these groups. Age disparities were adjusted for in the regression models, but the age differences between the persons with and without dementia need to be considered in the crude comparisons. Older patients may indeed be treated with older less expensive drugs than their younger counterparts [38].

## Conclusions

We found that the observed higher overall crude drug costs for persons with dementia were confounded by comorbidities and residential setting. Thus, it is important to include these confounders in pharmacoeconomic studies of dementia.

The highest drug cost was observed for nervous system drugs among persons with dementia and in institutions. Cardiovascular drugs were more commonly used by persons with dementia, but to a lower cost. Future studies should investigate whether these findings reflect appropriate care or an imperfect drug treatment among dementia patients.

## Abbreviations

CNS: Central nervous system
SNAC: Swedish National Study on Aging and Care
GLM: Generalized linear model
ATC: Anatomical Therapeutic Chemical
WHO: World Health Organization
DDD: Defined daily dose
ADL: Activities of daily living
SD: Standard deviation
ND: Non-dementia
D: Dementia
DMT: Disease modifying therapy
ACE: Angiotensin-converting enzyme
